# Prevalence and diagnostic ability of β-zone parapapillary atrophy in open-angle glaucoma: a systematic review and meta-analysis

**DOI:** 10.1186/s12886-022-02282-5

**Published:** 2022-02-12

**Authors:** Dengming Zhou, Mengdan Cao, Xuanchu Duan

**Affiliations:** 1 Department of Ophthalmology, The Second Xiangya Hospital, Central South University, Changsha, 410011 China; 2 Aier School of Ophthalmology, Central South University, Changsha, 410000 China; 3 Changsha Aier Eye Hospital, Aier Eye Hospital Group, Changsha, 410000 China

**Keywords:** OAG, β-zone parapapillary atrophy, Meta-analysis

## Abstract

**Background:**

β-Zone parapapillary atrophy (β-PPA) is a common sign in patients with open-angle glaucoma (OAG). Some studies have suggested that β-PPA can aid in the diagnosis of OAG. We performed a systematic review and meta-analysis of the prevalence and diagnostic ability of β-PPA in OAG.

**Methods:**

We performed a literature search in PubMed, Web of Science, Embase and Google Scholar from inception to 1st November, 2021. Both hospital-based and population-based studies that reported details of β-PPA in OAG were included.

**Results:**

We screened 1404 articles from these databases and ultimately included 24 articles in our meta-analysis. The prevalence of β-PPA in OAG was 0.73 (95% CI 0.67 to 0.78). The results of subgroup analysis by country revealed prevalence rates of 0.83 (95% CI 0.78 to 0.88) in Japan, 0.85 (95% CI 0.64 to 0.97) in Korea, 0.64 (95% CI 0.55 to 0.73) in the USA, 0.61 (95% CI 0.58 to 0.63) in Germany and 0.57 (95% CI 0.39 to 0.74) in China. Fundus photography, Heidelberg retina tomography (HRT), Heidelberg retina angiography (HRA) + indocyanine green angiography (ICGA), Spectral domain optical coherence tomography (SD-OCT)and Swept source optical coherence tomography(SS-OCT) values were 0.65 (95% CI 0.58 to 0.71), 0.70 (95% CI 0.50 to 0.86), 0.78 (95% CI 0.61 to 0.91), 0.77 (95% CI 0.65 to 0.88) and 0.99(95% CI 0.87 to 1.00) respectively. The sensitivity and specificity of β-PPA as a diagnostic marker were 0.78 (95% CI 0.68 to 0.85) and 0.63 (95% CI 0.51 to 0.73), respectively.

**Conclusions:**

β-PPA is a potential diagnostic marker for OAG. However, a more detailed understanding of β-PPA characteristics is needed to improve the ability to predict OAG.

**Supplementary Information:**

The online version contains supplementary material available at 10.1186/s12886-022-02282-5.

## Background

As we all know, glaucoma is a kind of irreversible blinding eye disease. The key to the treatment of glaucoma lies in early diagnosis, Now we diagnose it by the thickness of RNFL, vertical cup and disc ratio and Blood flow density in optic papilla. Of course, visual field examination is the most important. To be radical, it is too late when you are clinically diagnosed with glaucoma by the typical visual field defect. Therefore, it is of great significance to find a marker for early or auxiliary diagnosis of glaucoma. Parapapillary atrophy (PPA), which is also called halo glaucomatosus, was first discovered in patients with advanced glaucoma, and the relationship between parapapillary atrophy and glaucoma has long been recognised [1]. Jonas et al. [2] subdivided PPA into α-zone and β-zone PPA according to the results of fundus photography. The α-zone PPA is a region with irregular hypopigmentation or hyperpigmentation of the retinal pigment epithelium (RPE) and is located in the periphery of the PPA. The β-zone PPA is a region in which the RPE has disappeared and the choriocapillaris has atrophied [3]. In fundus photography, the β-zone PPA is characterised by visible sclera and large choroidal vessels located between the optic disc and the α-zone. Many studies have suggested that β-PPA could be a potential diagnostic marker for OAG [1–3]. With the development of techniques for obtaining fundus images, more detailed characteristics of β-PPA have been identified by fundus photography, confocal scanning laser ophthalmoscopy (such as Heidelberg retina tomography, HRT) and optical coherence tomography (OCT).

Recent research has shown that β-zone PPA can be used to diagnose OAG, with an area under the receiver operating characteristic (AUROC) curve value (95%) of 0.75 [4]. Here, we performed a systematic review and meta-analysis of the prevalence of β-PPA and its diagnostic ability in OAG. The purpose of this study is to investigate and objectively state the incidence of β-PPA in glaucoma and verify its diagnostic efficacy for further research.

## Methods

### Search strategy

We searched PubMed, Web of Science, Embase and Google Scholar for observational studies that reported the prevalence of β-PPA with and without a control group between inception and 1^st^ November, 2021. See “Additional file 1” for specific search strategy.

Our search terms were ‘((glaucoma, open-angle) OR (open-angle Glaucoma) OR (POAG) OR (OAG) OR (normal tension glaucoma) OR (NTG)) AND ((parapapillary chorioretinal atrophy) OR (parapapillary atrophy) OR (peripapillary chorioretinal atrophy) OR (peripapillary atrophy) OR (halo glaucomatosus))’.

### Eligibility criteria

The inclusion criteria were as follows: OAG patients who had been diagnosed comprehensively by perimetry, gonioscopy and intraocular pressure (IOP); β-PPA analysed on fundus photography images; the prevalence of β-zone PPA in OAG eyes could be determined or calculated from the article; and only cross-sectional studies were included. Studies were excluded according to the following criteria: studies that did not distinguish the α-zone and β-zone; those reporting only the area data of the β-zone without numbers or prevalence; studies in monkeys or that were not performed in vivo; studies that concentrated on the optic disc and not the whole parapapillary zone; and those for which the full text was not available.

### Data extraction

Two reviewers (DMZ and MDC) independently screened all titles and abstracts of articles and selected those that met the eligibility criteria. We read the full text of selected articles. Then, we chose articles based on the selection criteria for our systematic review and meta-analysis. If we had different opinions on including or excluding the study, a final decision was made by the lead author, Prof. Duan.

#### Prevalence of β-PPA

We extracted the number of OAGs with and without β-zone PPA and calculated the prevalence from all studies. We recorded the country, age, sex and diagnostic type.

#### Diagnostic ability of β-PPA

We selected articles from 24 studies with healthy individuals as controls or that were population-based. Then, a 2 × 2 table, i.e. true positives (TP), false positives (FP), true negatives (TN) and false negatives (FN), was generated from the selected studies. Diagnostic analysis according to TP, FP, FN and TN was also performed using Review Manager V5.3.

### Quantitative analysis

Data analysis was performed using R (version 3.5.0, R Foundation for Statistical Computing, Vienna, Austria) and Excel (Microsoft Redmond, WA, USA). Review Manager V5.3 was used for quality evaluation and diagnostic meta-analysis of β-PPA. Heterogeneity analysis and subgroup analysis of proportions were assessed using the meta package (4.9–2) in R. The proportions were logit transformed to fit the normal distribution. The meta-analysis model was chosen based on the I^2^. We performed subgroup analysis according to country and device.

## Results

### Selected studies

**Fig. 1:**
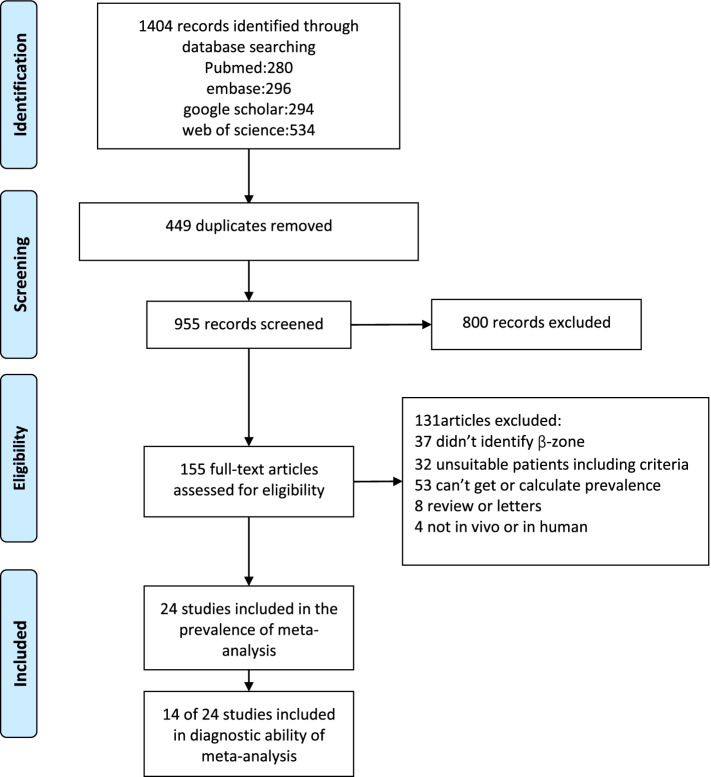
Flow diagram of study inclusion in the meta-analysis

Twenty-one studies were included in the proportion analysis [3, 5–24] and 14 studies were included in the diagnostic meta-analysis. We initially obtained 1404 articles from PubMed, Embase, Web of Science and Google Scholar. After the screening stage, 155 articles were passed into the eligibility stage (449 duplications were excluded and 800 articles were identified by reading the title and abstract). After reading the full text of these 155 articles, 24 articles were included in our meta-analysis. Figure 1 shows the detailed process and results of inclusion.

### Risk of bias

**Fig. 2:**
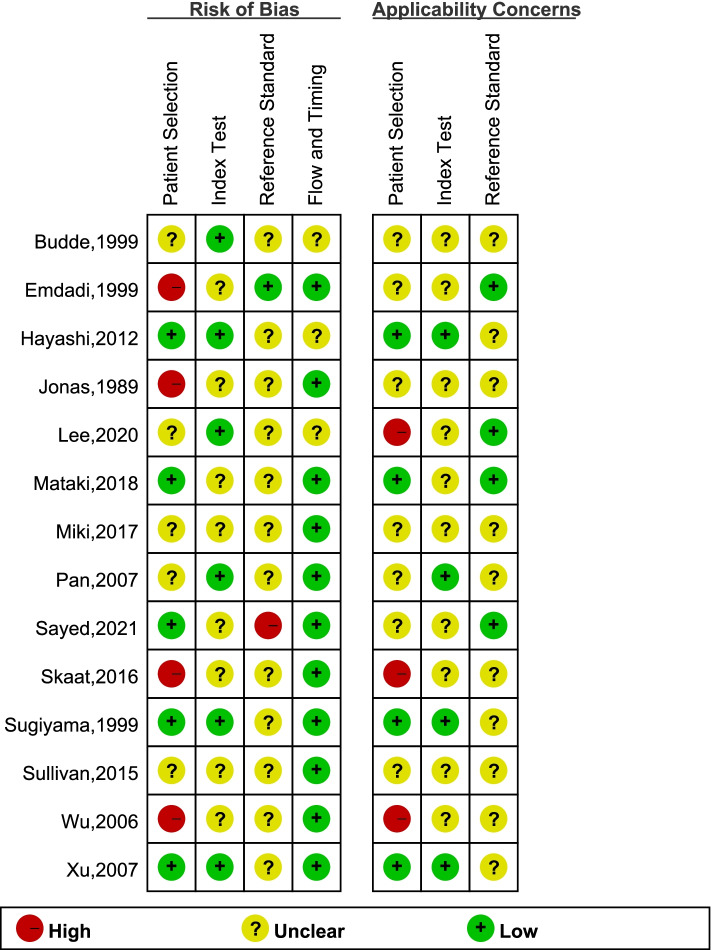
Twenty-one studies were subject to quality evaluation based on specific criteria

Quality evaluation of 24 studies was applied based on the Newcastle–Ottawa Scale for Cross Sectional Studies criteria (Supplement Table 1). The 14 of 24 studies were also assessed using the QUADAS-2 scale (Fig. 2). Here, I^2^ > 50% was commonly noted in the heterogeneity evaluation of prevalence and diagnostic ability. Thus, we choose the random effects model. Publication bias was analysed and is presented as a funnel diagram. Linear regression tests of funnel plot asymmetry used a random effects model: t = 0.88034, df = 22, *p* = 0.3882. (Supplementary Fig. 1).

**Table 1:** Characteristics of included studies

Study	Country	Prevalence	Quantity (eyes)	Diagnostic criteria	Age range (years)	Image	Refraction (D)	Study design
Jonas et al. [2, 24]	Germany	59.6%	582	Chronic OAG	62.9 ± 13.3	disc camera	0.2 ± 2.6	Hospital-based
Park et al. [23]	Japan	84.3%	102	NTG	57.8 ± 13.4	HRT	− 0.3 ± 2.3	Hospital-based
Tezel et al. [22]	USA	67.2%	529	POAG + NTG	68.4 ± 12.6	disc camera	Range, 3 to 3	Hospital-based
Budde and Jonas [21]	Germany	62.3%	501	POAG	62.7 ± 14.7	disc camera	− 0.4 ± 2.4	Hospital-based
Emdadi et al. [20]	USA	44.8%	29	POAG	62.3 ± 13.5	HRT	Range, 3 to 3	Hospital-based
Kono et al. [19]	USA	48.9%	47	POAG	63.4 ± 13.9	HRT	Range, 3 to 3	Hospital-based
Sugiyama et al. [18]	Japan	79.2%	207	OAG	48.1 ± 9.4	Fundus photography	NA	Population-based
Park et al. [17]	Korea	97.0%	33	NTG	48.0 ± 9.1	HRT	< − 6	Hospital-based
Budde and Jonas [16]	Germany	58.9%	168	Chronic OAG	55.8 ± 11.4	Fundus photography	− 1.2 ± 2.7	Hospital-based
Duan et al. [15]	China	39.8%	128	POAG	49.0 ± 15.6	Fundus photography	− 1.1 ± 2.4	Hospital-based
Wu et al. [13]	China	77.8%	27	POAG	63. 0 ± 13. 1	HRA and ICGA	− 0.9 ± 2.4	Hospital _based
Pan et al. [14]	China	43.5%	85	POAG + NTG	60.7 ± 11.0	Fundus photography	− 0.3 ± 1.0	Hospital _based
Xu et al. [12]	China	68.8%	93	Glaucoma	63.7 ± 10.1	Fundus photography	− 0.5 ± 2.4	Population-based
Teng et al. [11]	USA	59.5%	245	OAG	70.0 ± 12.3	HRT	− 1.0 ± 2.4	Hospital-based
Lee et al. [10]	Korea	71.3%	202	POAG + NTG	63.4 ± 11.3	Fundus photography	− 0.3 ± 2.1	Hospital-based
Hayashi et al. [9]	Japan	84.0%	100	POAG	55.6 ± 11.0	SD-OCT	− 2.2 ± 2.2	Hospital-based
Kim et al. [8]	Korea	79.0%	195	POAG	51.4 ± 13.9	SD(EDI)-OCT	− 2.7 ± 3.8	Hospital-based
Sullivan-Mee et al. [7]	USA	76.2%	63	POAG	67.2 ± 8.2	SD-OCT	AL:24.41 mm	Hospital-based
Skaat et al. [6]	USA	77.2%	801	Glaucoma	61.4 ± 12.8	Fundus photography	− 0.5 ± 1.9	Hospital-based
Miki et al. [3]	Japan	94.0%	50	OAG	59.8 ± 9.5	SS-OCT	− 2.4 ± 2.3	Hospital-based
Mataki et al. [5]	Japan	77.4%	84	POAG	63.9 ± 12.4	Fundus photography	− 0.9 ± 2.7	Population-based
Lee et al.[33]	Korea	100.0%	88	OAG	53.9 ± 13.4	SS-OCT	− 0.66 ± 2.16	Hospital-based
Lee et al.[34]	Korea	50.6%	77	Glaucoma	56.1 ± 12.7	SD-OCT	AL: 24.7 ± 1.6 mm	Hospital-based
Sayed et al.[35]	Egypt	90.0% (82%)	100	POAG	51.0 ± 8.8	SD-OCT (Fundus photography)	NA	Hospital-based

*Abbreviations*: *EDI-OCT* enhanced depth imaging OCT, *HRA* Heidelberg Retinal angiography, *HRT* Heidelberg retina tomography, *ICGA* indocyanine green angiography, *NTG* normal tension glaucoma, *OAG* open-angle glaucoma, *OCT* optic coherent tomography, *POAG* primary open-angle glaucoma, *SD-OCT* spectral-domain OCT, *SS-OCT* swept-source OCT

### Study characteristics

Table 1 presents the age, sex, country and refraction values of the patients included in these studies. We listed or calculated the prevalence from the included studies. Of all 24 studies, including 4,536 samples, three studies were performed in Germany, three in Korea, four in China, five in Japan and six in the USA. The mean age of the patients was 49–70 years. Most [11] studies used fundus photography to obtain images, five used Heidelberg retinal tomography (HRT), three used optic coherence tomography (OCT) and one used both Heidelberg retinal angiography and indocyanine green angiography (ICGA). Three articles described population-based studies and the others reported hospital-based studies. All studies were cross-sectional or retrospective in design.

### Prevalence

**Fig. 3:**
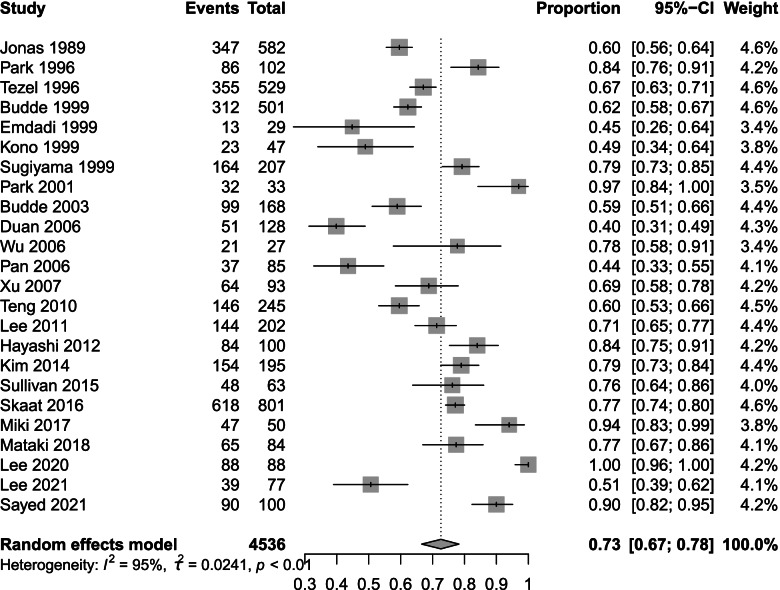
Total prevalence in 24 studies. The total prevalence of 4,536 included eyes was 73% (95% CI 67 to 78) based on a random effects model

**Fig. 4:**
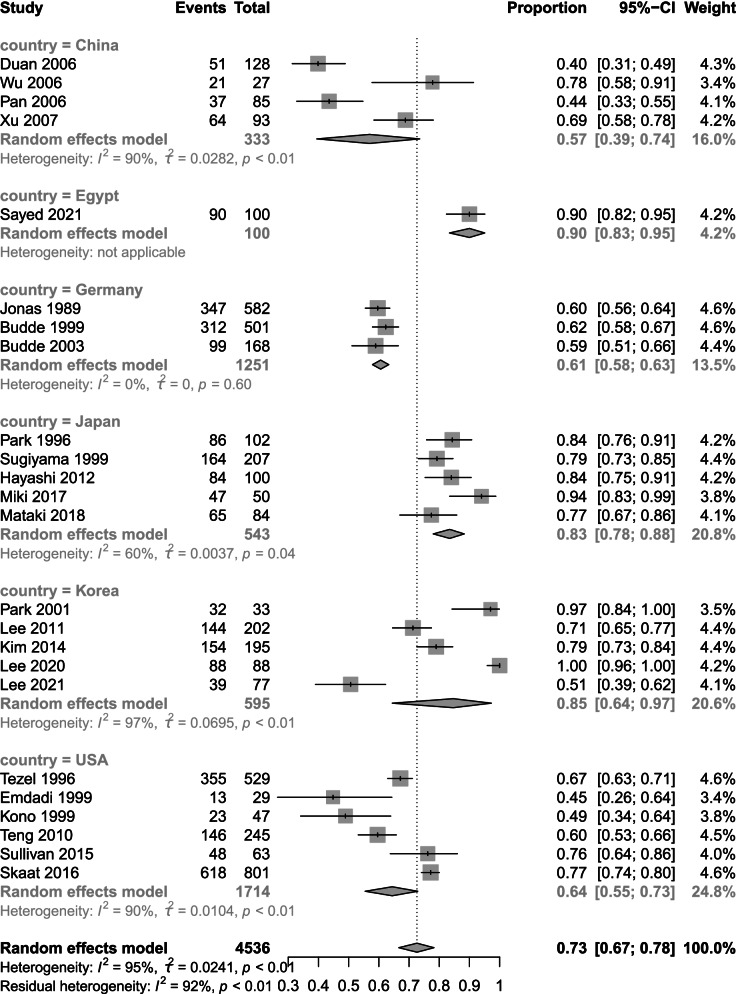
Subgroup analysis based on country. The prevalence rates were 57% (95% CI 39 to 74) in China, 61% (95% CI 58 to 63) in Germany, 83% (95% CI 78 to 88) in Japan, 85% (95% CI 64 to 97) in Korea and 64% (95% CI 55 to 73) in the USA

**Fig. 5:**
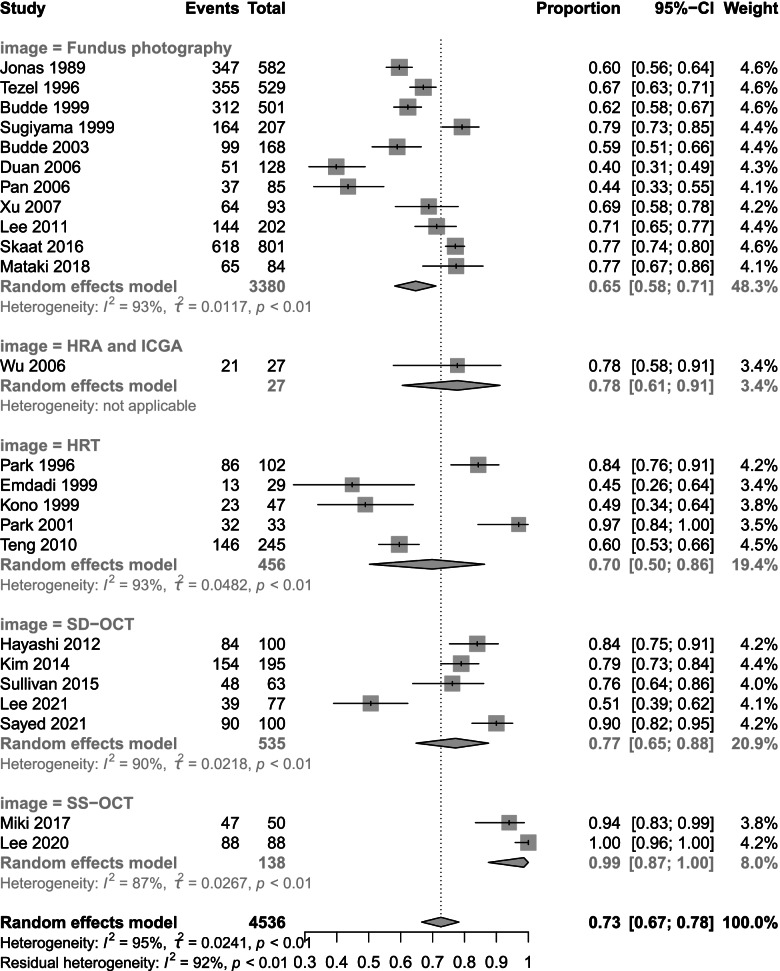
Subgroup analysis based on the imaging method. Fundus photography exhibited the lowest prevalence of 65% (95% CI 58 to 71) followed by HRT at 70% (95% CI 50 to 86), SD-OCT at 77% (95% CI 65 to 88) and HRA + ICGA at 78% (95% CI 61 to 91). SS-OCT had the highest prevalence at 99% (95% CI 87 to100)

We analysed the prevalence data and chose logit transformation to obtain a normal distribution. The I^2^ value was 95%, τ^2^ = 0.0241 and *p* < 0.001. Based on the I^2^ values, we chose a random effects model. The prevalence of β-PPA in OAG was 73% (95% CI 67 to 78) (Fig. 3). The results of subgroup analysis by country and device are shown below. Subgroup analysis by country showed prevalence rates of 57% (95% CI 39 to 74) in China, 61% (95% CI 58 to 63) in Germany, 83% (95% CI 78 to 88) in Japan, 85% (95% CI 64 to 97) in Korea and 64% (95% CI 55 to 73) in the USA (Fig. 4). Subgroup analysis by device revealed differences in prevalence rate among fundus photography, HRT, HRA + ICGA and OCT. Fundus photography showed the lowest prevalence of 65% (95% CI 58 to 71) followed by HRT at 70% (95% CI 50 to 86) SD-OCT 77% (95% CI 65 to 88) and HRA + ICGA at 78% (95% CI 61 to 91). SS-OCT had the highest prevalence at 99% (95% CI 87 to 100) (Fig. 5).

### Diagnostic ability

**Fig. 6:**
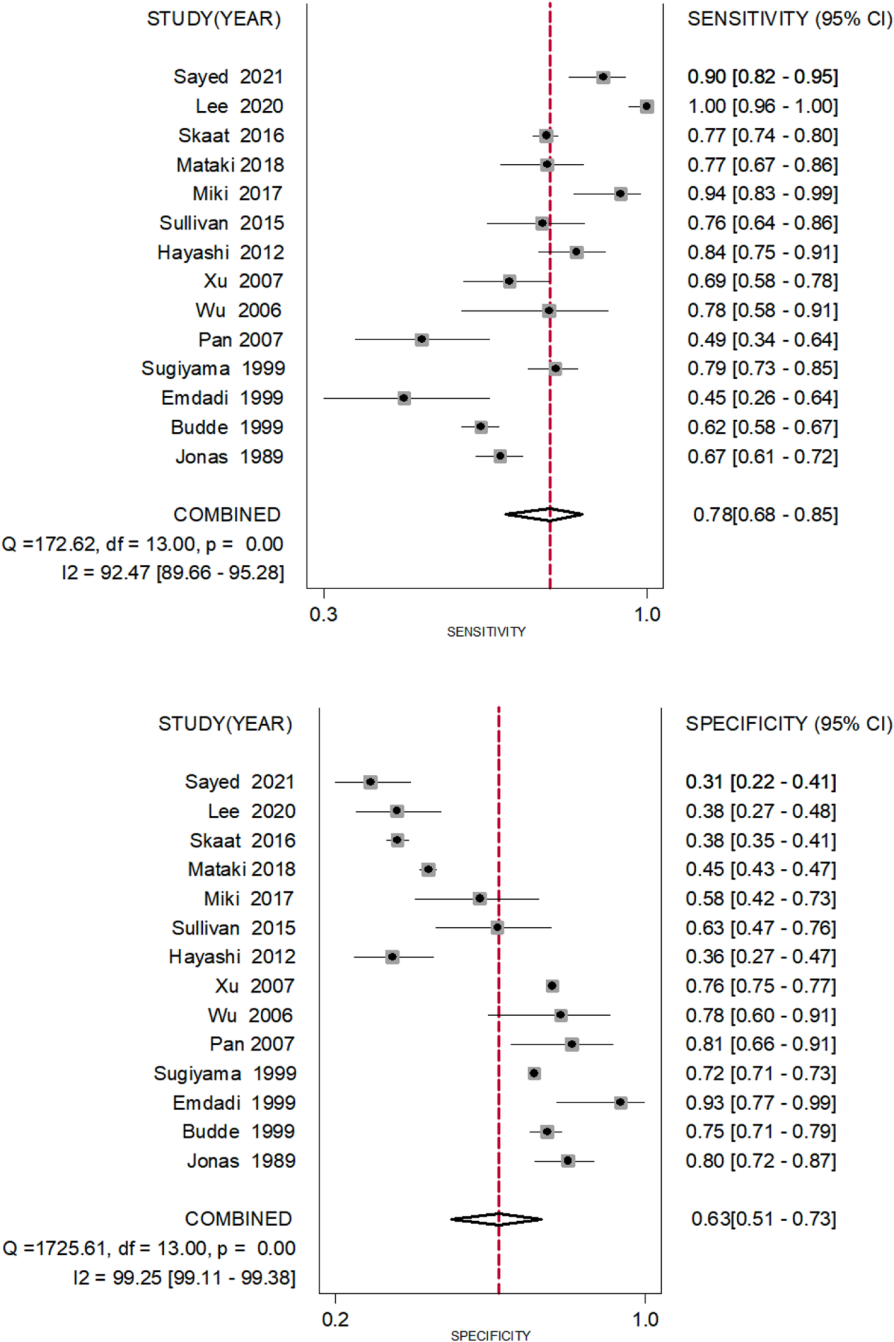
The sensitivity and specificity of β-PPA as a single diagnostic biomarker. The sensitivity to distinguish true positive for glaucoma was 0.78 (95% CI 0.68 to 0.85); the specificity to distinguish true negative for glaucoma was 0.63 (95% CI 0.51 to 0.73)

**Fig. 7:**
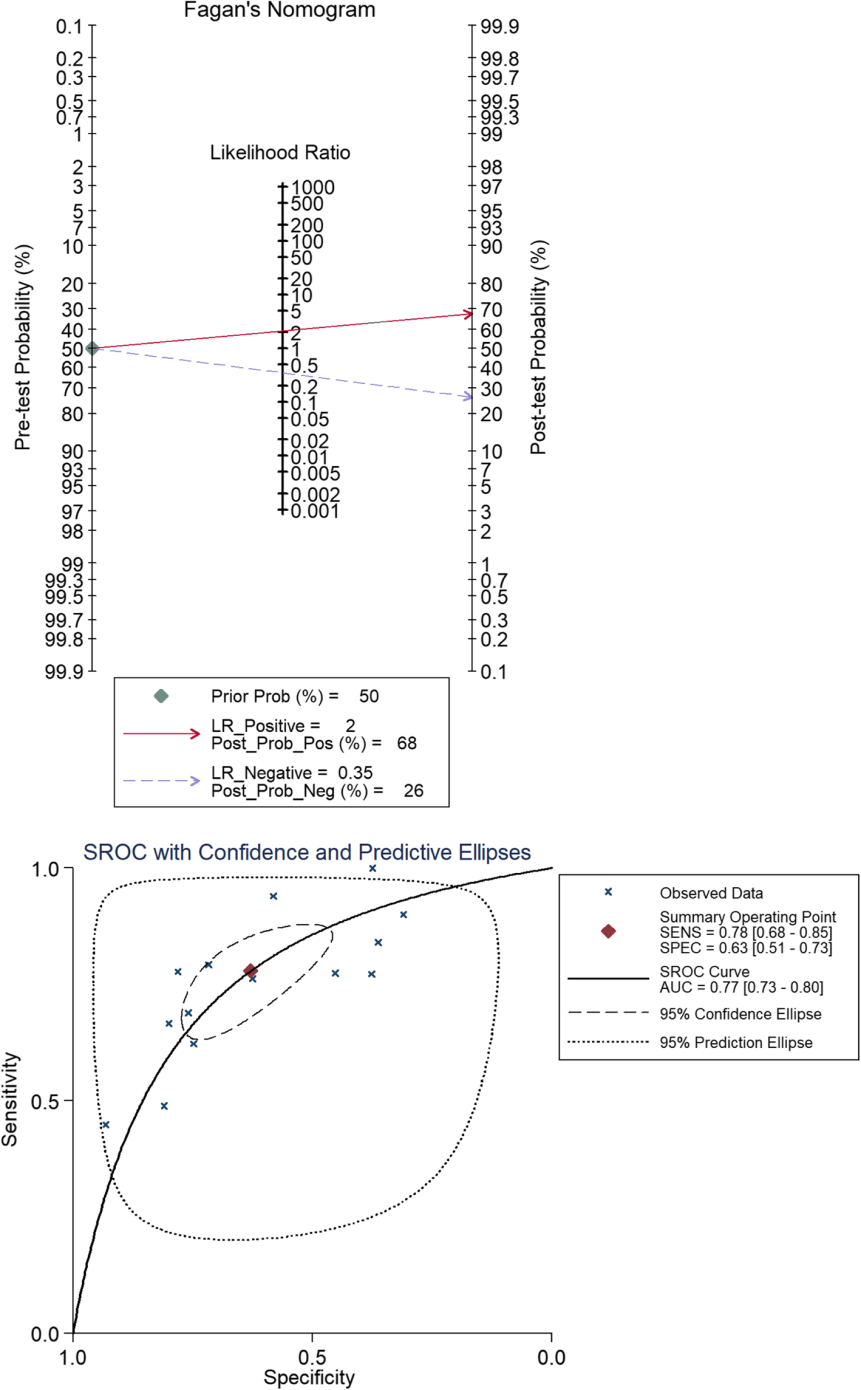
Fagan’s nomogram and SROC. The post-test probability positive percentage and post-test probability negative percentage were 68% and 26%, respectively, while the prior probability percentage was 50%. The area under the SROC(AUROC) curve was 0.77

We compiled 2 × 2 tables according to 14 studies of 24 included articles a shown in Table 2. Fig. 6 shows the sensitivity and specificity of β-PPA as a single diagnostic marker for glaucoma, which had sensitivity of 0.78 (95% CI 0.68 to 0.85) and specificity of 0.63 (95% CI 0.51 to 0.73) (Fig. 6). The diagnostic index was 141%. We generated a Fagan figure and symmetric receiver operator characteristic (SROC) curve. According to the Fagan figure, 50% of patients were diagnosed with glaucoma. The possibility increased to 68% in patients with β-PPA, and decreased to 26% in those without β-PPA. The area under the SROC (AUROC)curve was 0.77 (Fig. 7).

**Table 2:** Characteristics of studies assessing diagnostic ability of β-PPA

Study	Numbers of POAG	Numbers of healthy controls	Index test	TP	FP	FN	TN
Jonas et al. [2, 24]	312	125	Clinical diagnosis	208	25	104	100
Budde and Jonas [21]	501	481	Clinical diagnosis	312	121	189	360
Emdadi et al. [20]	29	29	Clinical diagnosis	13	2	16	27
Sugiyama [18]	207	11,727	Glaucomatous optic neuropathy	164	3319	43	8408
Pan et al. [14]	45	42	Clinical diagnosis	22	8	23	34
Wu et al. [13]	27	32	Clinical diagnosis	21	7	6	25
Xu et al. [12]	93	3910	Clinical diagnosis	64	938	29	2972
Hayashi et al. [9]	100	100	Clinical diagnosis	84	63	16	36
Sullivan-Mee et al. [7]	63	48	Clinical diagnosis	48	18	15	30
Miki et al. [3]	50	43	Clinical diagnosis	47	18	3	25
Mataki et al. [5]	84	2129	Clinical diagnosis	65	1165	19	964
Skaat et al. [6]	801	1149	Glaucomatous optic neuropathy	618	716	183	433
Lee et al.[33]	88	88	Clinical diagnosis	88	55	0	33
Sayed et al.[35] (SD-OCT)	100	100	Clinical diagnosis	90	69	10	31

Clinical diagnosis means that ‘POAG’ & ‘healthy controls’ were diagnosed not only by morphology of the optic disc but also by other clinical symptoms, including visual field, IOP and thickness of optic nerve fibre layer. Glaucomatous optic neuropathy means that the researchers only studied the morphology of glaucomatous optic discs

*Abbreviations*: *FN* false negative, *FP* false positive, *TN* true negative, *TP* true positive

Sensitivity indicates that the TP rate is equivalent to TP/(TP + FN). Specificity indicates that the TN rate is equivalent to TN/(TN + FP)

## Discussion

As glaucoma leads to irreversible blindness, there is a great deal of interest in means to improve the diagnostic ability of OAG. The primary OAG preferred practice pattern guidelines [25] state that parapapillary atrophy is one of the physical features indicating glaucomatous optic neuropathy. The development of parapapillary choroidal atrophy in early glaucoma may precede the onset of visual field defects [26]. According to the definition of β-PPA, logically speaking, Its diagnostic criteria for glaucoma have anatomical and physiological basis. There are no RPE cells in β-PPA. However RPE plays an irreplaceable protective role in retina 0.1.RPE cells absorb light, reduce oxidative damage of other cells. 2.RPE cells maintain strong transport function and local acid–base and ion balance. 3.In addition, it can also provide a variety of neurotrophic substances, such as bFGF, HGF, etc. All in all,whether RPE cell damage is the initial factor or not, ganglion cells in this region without RPE are more vulnerable finally.

This is the first systematic review of the prevalence of β-PPA in OAG eyes. Our meta-analysis of 24 studies indicated that the prevalence of β-PPA in glaucoma patients is very high up to 70%). Subgroup analysis by country revealed different prevalence rates in different countries, with Korea and Japan showing the highest prevalence. This may be related to the high incidence of high myopia in East Asian populations. With elongation of the ocular axis, the uvea of the eyeball becomes thinner, thus increasing the appearance of β-zone PPA. The USA and Germany showed similar prevalence rates of approximately 64%. Skaat reported that Africans with glaucoma exhibited an increased prevalence of β-zone PPA compared with Europeans [6], suggesting that there may be structural differences within the optic nerve complex associated with ethnicity. In contrast, China had the lowest prevalence. It is difficult to explain the observed differences based on race. Further studies in Chinese populations are needed for further analysis. Continued development of fundus imaging technology will allow easier and more accurate confirmation of β-zone PPA. Studies using OCT had the highest prevalence followed by HRA, HRT and fundus photography. This order can be understood easily as fundus photography produces only a two-dimensional image, while OCT provides not only en face images but also B-scans from which more precise and comprehensive parapapillary information can be obtained.

OAG showed passable diagnostic ability for β-zone PPA. However, for clinical use the diagnostic efficiency of this diagnostic tool must be improved. Our meta-analysis indicated several limitations regarding its efficiency. First, it will be necessary to solve the problem of the threshold effect. In this review and most studies, the threshold was ‘0’, indicating that we judged the presence or absence of the β-zone. Advanced OCT can easily distinguish the end point of the RPE, and we can precisely measure the area of the β-zone. It seems better to set a reasonably low limit value. As observed, the threshold values should be different for different races because of the ethnic differences in parapapillary structures. On the other hand, some surveys suggested that the first stage in the process of β-zone PPA progression is the loss of RPE cells, followed by the loss of photoreceptors and, finally, by closure of the chorion. In addition, Dai et al. [27] proposed a more detailed method to divide the β-zone into subregions. These groups referred to these areas as the β-zone and γ-zone, which are bounded by the end point of Bruch’s membrane (BM); some groups also refer to them as βBM + and βBM − , respectively. The β-zone/βBM + was also shown to be associated with glaucoma, while the γ-zone/ βBM − was associated with myopia [3, 28, 29]. The subclassic β-zone is a promising novel tool for diagnosis of glaucoma with high myopia compared to the retinal nerve fibre layer (RNFL) and cup/disc (C/D) ratio. Therefore, another approach to increase the efficiency involves the use of the new subregion method. Refractory/axial length and age may be confounding factors [3, 30–32]; older people or those with longer axial lengths are more likely to have β-zones or larger regions. The threshold should be corrected in these patients based on Big Data analytics. Our analysis also showed that regardless of the observation method used in research after 2010, the sensitivity was greatly improved, while the specificity decreased. This may be due to the improvement of identification of the β-zone by clinicians. Thus, the β-zone has great potential as a negative indicator (high sensitivity and low specificity), i.e. glaucoma can be excluded based on the lack of a β-zone. For example, in the clinic, we often encounter patients with a physiological large optic cup (usually teenagers). When it is not easy to make a judgement based on the shape of the optic disc, if the β-zone is negative, glaucoma can be excluded in these cases. However, only three of the 12 studies included in the meta-analysis were population-based studies (i.e. Sugiyama [18], Xu [12] and Mataki [5]), meaning that our analysis made use of publications describing populations that were enriched for the presence of glaucoma. This would have led to overestimation of the sensitivity of this diagnostic tool. In conclusion, β-PPA is a potential diagnostic marker for OAG. However, a more detailed understanding of the characteristics of β-PPA is needed to improve the ability to identify and predict OAG. The prevalence of β-PPA differs between countries, with a higher incidence in Korea and Japan than in the USA and Germany. The prevalence of β-PPA was also shown to differ according to the method used to obtain images, with the best method being OCT, especially swept-source OTC (SS-OCT). Despite the general ability of β-PPA to diagnose glaucoma, setting an eligible threshold using a more advanced partition method and appropriate correction for race, age and axial length represents a novel and effective approach.

## Conclusions

β-PPA is an important potential index for the diagnosis and prognosis of OAG. However, We need more data to define β-PPA precisely through the use of new fundus image acquisition method. More detailed understanding of β-PPA characteristics is needed to improve the ability to predict OAG.

## Supplementary Information

**Additional file 1. **

**Additional file 2. **

**Additional file 3. **

## Data Availability

Not applicable

## Abbreviations

β-PPA: β-Zone parapapillary atrophy
OAG: Open-angle glaucoma
NTG: Normal tension glaucoma
POAG: Primary open-angle glaucoma
HRT: Heidelberg retina tomography
HRA: Heidelberg retina angiography
ICGA: Indocyanine green angiography
OCT: Optical coherence tomography
SD-OCT: Spectral-domain OCT
EDI-OCT: Enhanced depth imaging OCT
SS-OCT: Swept-source OCT
