# Supplementary material for: Prevalence and diagnostic ability of β-zone parapapillary atrophy in open-angle glaucoma: a systematic review and meta-analysis
Source: BMC Ophthalmol. 2022 Feb 12;22:72. doi: 10.1186/s12886-022-02282-5 (PMC8840052; doi:10.1186/s12886-022-02282-5)
Supplement: Supplementary file 1 — Additional file 1. [file 12886_2022_2282_MOESM1_ESM.docx]

**Appendix of formal search strategy**

**Search strategy On “pubmed”: ((Glaucoma, Open-Angle) OR (open angle Glaucoma) OR (POAG) OR (OAG) OR (normal tension glaucoma) OR (NTG)) AND ((Parapapillary chorioretinal atrophy) OR (Parapapillary atrophy) OR (Peripapillary chorioretinal atrophy) OR (Peripapillary atrophy) OR (Halo glaucomatosus))  Filters: from 1000/1/1 - 2020/7/10 Records:259**

**Search strategy On “embase”: ((Glaucoma, Open-Angle) OR (open angle Glaucoma) OR (POAG) OR (OAG) OR (normal tension glaucoma) OR (NTG)) AND ((Parapapillary chorioretinal atrophy) OR (Parapapillary atrophy) OR (Peripapillary chorioretinal atrophy) OR (Peripapillary atrophy) OR (Halo glaucomatosus))  Filters: from 1000/1/1 - 2020/7/10 Records:268**

**Search strategy On “google scholar”:** ((Glaucoma, Open-Angle) OR (open angle Glaucoma) OR (POAG) OR (OAG) OR (normal tension glaucoma) OR (NTG)) AND ((Parapapillary chorioretinal atrophy) OR (Parapapillary atrophy) OR (Peripapillary chorioretinal atrophy) OR (Peripapillary atrophy) OR (Halo glaucomatosus)) **Date:** 1900 to 2020 **Records:240**

**Search strategy On “web of sicence”:** ((Glaucoma, Open-Angle) OR (open angle Glaucoma) OR (POAG) OR (OAG) OR (normal tension glaucoma) OR (NTG)) AND ((Parapapillary chorioretinal atrophy) OR (Parapapillary atrophy) OR (Peripapillary chorioretinal atrophy) OR (Peripapillary atrophy) OR (Halo glaucomatosus)) **(Topic) Index Date:** 1900-01-01 to 2020-07-10 **Records:499**
