# Supplementary material for: Prevalence and diagnostic ability of β-zone parapapillary atrophy in open-angle glaucoma: a systematic review and meta-analysis
Source: BMC Ophthalmol. 2022 Feb 12;22:72. doi: 10.1186/s12886-022-02282-5 (PMC8840052; doi:10.1186/s12886-022-02282-5)
Supplement: Supplementary file 2 — Additional file 2. [file 12886_2022_2282_MOESM2_ESM.docx]

| **Newcastle-Ottawa Scale for Cross Sectional Studies** | | | | | | | | |
| --- | --- | --- | --- | --- | --- | --- | --- | --- |
| **Study** | **Selection** | | **Comparability** | | **Outcome** | | **Total Score** | |
|  | Representativeness of the sample | Sample  size | Non-respondents | Ascertainment of exposure | Risk  factors | Assessment of outcome | Statistical test |  |
| Jonas,1989 | * | * | * | ** | * | * | * | 8 |
| Park,1996 | * | / | * | ** | * | * | * | 7 |
| Tezel,1996 | / | * | * | * | ** | * | * | 7 |
| Budde,1999 | * | * | * | ** | * | * | * | 8 |
| Emdadi,1999 | * | / | / | * | ** | * | * | 6 |
| Kono,1999 | * | / | / | ** | ** | * | * | 7 |
| Sugiyama,1999 | * | * | * | ** | * | * | * | 8 |
| Park,2001 | * | / | / | ** | * | * | * | 6 |
| Budde,2003 | * | * | / | * | ** | / | * | 6 |
| Duan,2006 | * | / | / | ** | * | * | * | 6 |
| Pan,2006 | * | / | / | * | ** | * | * | 6 |
| Wu,2006 | * | / | / | ** | ** | * | * | 7 |
| Xu,2007 | * | * | * | ** | ** | * | * | 9 |
| Teng,2010 | * | * | / | * | ** | * | * | 7 |
| Lee,2011 | * | * | * | ** | ** | * | * | 9 |
| Hayashi,2012 | * | * | / | ** | ** | * | * | 8 |
| Kim,2014 | * | * | / | ** | ** | * | * | 8 |
| Sullivan,2015 | * | / | / | ** | ** | / | * | 6 |
| Skaat,2016 | * | * | * | ** | ** | * | * | 9 |
| Miki,2017 | * | / | * | ** | ** | / | * | 7 |
| Mataki,2018 | * | / | * | ** | ** | * | * | 8 |
| Lee,2020 | * | / | / | ** | ** | * | * | 7 |
| Lee,2021 | * | / | / | ** | ** | / | * | 6 |
| Sayed,2021 | * | / | / | ** | * | / | * | 5 |

Supplement Table 1: Each included study was critically appraised by using the Newcastle-Ottawa quality assessment scale for the modified version for cross-sectional studies. The scale assessed the studies for the following 3 items: 1) Selection; 2) Comparability; 3) Exposure/Outcome.
