# Supplementary figures and images for: Prevalence and diagnostic ability of β-zone parapapillary atrophy in open-angle glaucoma: a systematic review and meta-analysis
Source: BMC Ophthalmol. 2022 Feb 12;22:72. doi: 10.1186/s12886-022-02282-5 (PMC8840052; doi:10.1186/s12886-022-02282-5)

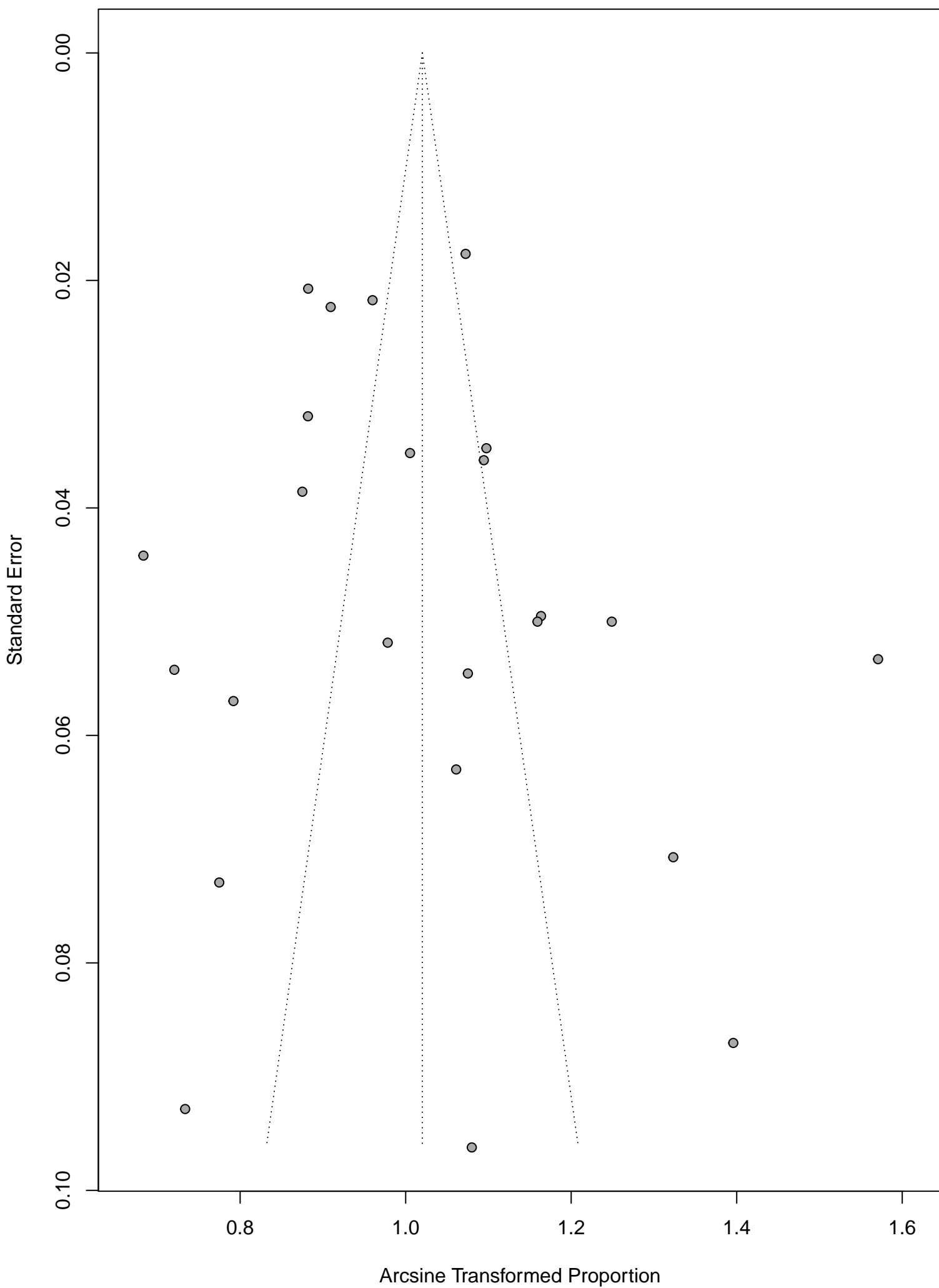

Supplement: Supplementary file 3 — Additional file 3. [file 12886_2022_2282_MOESM3_ESM.pdf]
